# A novel conceptual framework for balance training in Parkinson’s disease-study protocol for a randomised controlled trial

**DOI:** 10.1186/1471-2377-12-111

**Published:** 2012-09-27

**Authors:** David Conradsson, Niklas Löfgren, Agneta Ståhle, Maria Hagströmer, Erika Franzén

**Affiliations:** 1 Department of Neurobiology, Care Sciences and Society, Division of Physiotherapy, Karolinska Institute, 23100, SE-14183, Huddinge, Sweden; 2 Department of Physical Therapy, Karolinska University Hospital, Stockholm, Sweden

## Abstract

**Background:**

There is increasing scientific knowledge about the interaction between physiological (musculoskeletal, neuromuscular, cognitive and sensory) systems and their influence on balance and walking impairments in Parkinson’s disease. We have developed a new conceptual framework for balance training, emphasising specific components of balance control related to Parkinson’s disease symptoms by using highly challenging, progressive and varying training conditions. The primary aim of this proposed randomised controlled trial will be to investigate the short-term and long-term effects of a 10-week balance training regime in elderly with Parkinson’s disease.

**Methods/Design:**

Eighty participants with mild to moderate idiopathic Parkinson’s disease will be recruited and randomly allocated to an intervention group receiving balance training or a control group whose participants will continue to receive their usual care. The intervention will consist of a 10-week group training regime (1-hour training, three times per week), which will be led by two physiotherapists to ensure training progression and safety. The conceptual framework will be applied by addressing specific balance components (sensory integration, anticipatory postural adjustments, motor agility, stability limits) through varying training conditions and structured progression. Assessment will be conducted through a multi-dimensional battery of outcomes, prior to and immediately after the 10-week intervention, and at 9 and 15 months’ follow-up after entering the study. Primary outcome measures will be balance performance (assessed using the Mini Balance Evaluation Systems Test), change in gait velocity (m/s) between single and dual task walking, and fear of falling (evaluated using the Fall Efficacy Scale International).

**Discussion:**

This study has the potential to provide new insight and knowledge of the effects of specific, varied and challenging balance training on a wide health spectrum in elderly with PD. If found to be effective, this pragmatic approach with translation of theory into practice, can be implemented in existing outpatient care.

**Trial registration:**

NCT01417598

## Background

Parkinson’s disease (PD) is a major worldwide health problem [1], with a reported incidence rate ranging from 11 to 19/100,000 per year in the European population [2]. Balance and walking impairments are present even in the early stages of PD [3-6] and have been shown to be associated with restrictions in everyday activities [7] and reduced quality of life (QoL) [8].

Furthermore, elderly with PD have shown reduced levels of physical activity [9] and a nine times increased risk of injurious falls, compared with the healthy elderly [10]. As PD progresses, balance problems gradually increase and are generally non-responsive to or worsen with levodopa treatment [11-13]. Currently, there is a growing body of research that highlights the role of physical exercise as an essential part of managing the disease, with potential neuroprotective mechanisms [14,15]. The effects of balance training on balance and gait performance are promising for individuals with PD [16-18], although several questions remain unanswered, particularly regarding dose, intensity and duration, as well as regarding specific exercises to improve balance control in the different stages of the disease [17-21]. Additionally, there is a lack of knowledge of the long-term effects of training interventions on participation in everyday living, such as physical activity and QoL [16].

Balance control relies on the interaction of several physiological systems (the musculoskeletal, neuromuscular, cognitive and sensory systems) with environmental factors and the performed task [22]. Balance impairment or postural instability in individuals with PD is often associated with poor or absent *reactive responses* following external perturbations, such as performing a rapid step following a slip or trip [23]. However, as degeneration of the basal ganglia affects many physiological systems essential for balance control, balance disorders in PD cannot be addressed to one single function but, rather, are the result of impairments of multiple systems [24-26]. First of all, dysfunction of the basal ganglia influences *sensory integration*, i.e. the ability of the central nervous system to transform different modalities of sensory information (somatosensory, visual and vestibular) into a single reference frame, which is important for estimation of limb and body position in relation to the environment [27]. Particularly, individuals with PD have shown impaired proprioception [13,27], overestimation of the amplitude of movements [28] and over-reliance on vision for balance control [29]. Moreover, the central motor drive has shown to be impaired in PD [30], causing bradykinetic movements and poorly timed and scaled *anticipatory postural adjustments* (APAs), i.e. feedforward control to stabilise the posture before and during voluntary movements [31,32]. Furthermore, the PD-characteristic “stooped” posture, decreased joint range of motion, narrow foot stance and axial rigidity increase the risk of instability since these musculoskeletal constraints reduce the *functional limits of stability*, i.e. the area in which the body centre of mass (CoM) can be moved with maintained stability and without changing the base of support (BoS) [33,34]. Taken together, sensory-motor interaction is essential for balance control and consequently, as discussed by Konczak et al. [27], sensory problems may degrade movement responses and cause instability to individuals with PD.

The functions of the basal ganglia incorporate motor program selection and adaptation [35,36], here referred to as *motor agility*, which involves maintenance of coordination between body parts, task-specific adjustments of movement and quick shifts from one task to the next. Deficient motor regulation in PD, manifested as poor inter-segmental coordination [37], problems adopting postural synergies [35,38] and delayed change of motor commands when shifting from one task to another [39], is believed to contribute to increased instability and freezing of gait during fall-related activities [40,41]. Another critical aspect of balance control in PD is the ability to divide attention and simultaneously process multiple tasks (motor or cognitive), i.e. *multi-tasking.* While performing a multi-task activity, individuals with PD compared with healthy subjects are more inclined to shift attention away from the balance task, which can lead to falls [24].

To be successful, all types of training require the application of basic training principles of (i) *specificit*y, i.e. being specific to the targeted function; (ii) *progressive overload*, i.e. providing a challenging overload to the physiological system through a certain level of intensity and regularity; and (iii) *varied practice*, i.e. promoting variation between exercise conditions [42,43]. To be specific, balance training needs to target functions, or impairments, of balance control associated with PD symptoms. As previously mentioned, there is increasing scientific knowledge concerning the interaction between and impact of different physiological systems for balance disorders in PD [24-26]. Previous intervention studies targeting balance performance in PD have, however, mainly emphasised non-specific training or compensatory training [17,44]. Moreover, to stimulate relearning of physiological systems important for balance control, the challenge level (i.e. intensity of the training, the difficulty level of balance exercises and the total training dose) needs to be considered. Allen et al. [17] recently concluded, based on a meta-analysis of exercise interventions targeting balance performance in PD, that previous studies in general have used low challenge levels, suggesting that future exercise interventions should focus on more challenging aspects of balance exercises and a higher training dose. Furthermore, to enhance generalisation of learned motor skills to a wide variety of situations and promote a multi-faceted repertoire of movement strategies, it is essential to guarantee practice through a wide variety of balance exercise conditions [43].

We hypothesise that a new conceptual framework for balance training, emphasising critical aspects of balance control specifically related to PD symptoms through highly challenging, progressive and varied exercises, will improve, or maintain, balance and gait performance in elderly with PD. Moreover, we predict that increased physical functioning following training will lead to greater confidence in everyday participation, improved QoL and increased levels of physical activity.

The primary aim of this proposed randomised controlled trial will be to expose elderly individuals with mild to moderate PD to a 10-week balance training regime, and compare the efficacy of balance training to care as usual using laboratory, clinical, free-living and self-perceived assessments before and immediately after the intervention as well as 9 and 15 months post-baseline.

## Methods

### Design

In a prospective, randomised controlled clinical trial, with 9 and 15 months’ post-baseline follow-up, 80 elderly patients with PD living in Stockholm County, Sweden, will be included. The project has been approved by the Regional Board of Ethics in Stockholm (Dnr: 2009/819-32 and 2010/1472-32).

### Participant selection

Community-dwelling individuals with PD will be recruited from Karolinska University Hospital, the Swedish Parkinson Associations and outpatient neurological clinics as well as by physicians with a specialty in neurology in Stockholm County. Advertisements in local newspapers will also be used to recruit participants. Potential participants will receive written information about the aims of the study and its procedure, with an invitation to indicate their interest in participating to the study coordinator. Inclusion criteria will be a clinical diagnosis of “idiopathic” PD, according to the Queens Square Brain Bank criteria, ≥60 years of age, have adapted to their present PD-medication and the ability to independently ambulate indoors without a walking aid. We will include mild to moderate PD (Hoehn and Yahr scores II and III) [45], since physical group training has been proved feasible for this level of disease severity [46,47]. Exclusion criteria will be a history suggesting “atypical” PD symptoms, as defined by Hughes et al. [48], a Mini-Mental State Examination (MMSE) score ≤24 [49] and other existing neuromuscular disorders or medical conditions that substantially influences their gait and balance performance or their participation in the training program. To screen potential participants’ eligibility, a physiotherapist will conduct a telephone interview based on a clinical self-report form covering questions about personal and medical history and self-perceived balance performance.

### Randomisation

Participants will be divided into two geographic cohorts (south and north) to reduce the travelling time for the participants. After baseline assessments, the participants in each cohort will be randomly assigned to two different groups in blocks of four, one experimental group with balance training and one control group. The sequence of allocation will be performed using web-based software and will be concealed to the participants and testers until the baseline measurement is completed. Blinding of group allocation will, however, not be possible since the test leaders will be supervising the training intervention (as described below).

### Intervention group

We have applied the training principles of specificity, varied practice and progressive overload to design a conceptual framework for balance training in PD. The framework comprises specific exercises associated with balance and walking constraints in PD (Table 1). The intervention period is divided into three blocks (A, B and C) to promote appropriate progressive overload through the course of training (Table 2). Implementation of the training principles for balance training and motor learning is outlined below.

**Table 1:** **Parkinson’s disease (PD) specificbalance components, constraints affectingbalance and exercises designedto reduce these constraints**

**Balance components**	**Constraints in PD**	**Exercise principles**	**Exercise objectives**
**SENSORY INTEGRATION**			
Integration of sensory information (somatosensory, visual and vestibular) for estimation of body position	- Impaired somatosensory integration	Walking tasks on varying surface with or without visual constraints	Improve interpretation of and reliance on somatosensory information
	- Poor proprioception		
	- Visual dependency		
**APAs**			
Prediction and control of perturbation related to voluntary movements	- Poorly timed and scaled APAs	Voluntary arm/leg/trunk movements focusing on movement velocity and amplitude, and postural transitions	Improve APA strategies regarding quality (timing, amplitude) and task- specific adaptation
	- Bradykinesia		
**MOTOR AGILITY**			
Coordination between body parts and movement adaptation, e.g. regulation of movement and quick shifts between tasks	- Bradykinesia	Whole-body coordination during varying gait conditions and reciprocal movements. Quick shifts of movement characteristics (velocity, amplitude and direction) during predictable and unpredictable conditions	Improve whole-body coordination, ability to adapt movement and quick shifts between different tasks
	- Impaired whole-body coordination		
	- Biomechanical constraints		
	- Inflexible motor programming		
**STABILITY LIMITS**			
Whole-body regulation relative to the BoS	- Reduced functional stability limits	Voluntary leaning tasks in standing with varying BoS-stimulating weight shifts in multiple directions through arm and trunk movements	Improve the ability to safely control CoM within BoS to increase functional limits of stability
	- Biomechanical constraints		
	- Poor proprioception		
	- Impaired somatosensory integration		

APAs = anticipatory postural adjustments; BoS = base of support; CoM = centre of mass.

**Table 2:** Training progression

**Training principles and objectives**		**Week**	**Balance components**	**Multi-task**
**A**	Introduction of performance of each balance component separately and emphasizing quality of performance to accomplish familiarity and task-specific motor learning.	**1**	Motor agility/stability limits	
		**2**	Sensory integration/APAs	
**B**	Improvement of balance performance and strategies of attention in varying balance conditions through increased level of difficulty and task variation for each balance component separately, and by using multi-tasking (i.e. cognitive or motor secondary task).	**3**	Motor agility/stability limits	C-DT
		**4**	Sensory integration/APAs	M-DT
		**5**	Motor agility/stability limits	C-DT
		**6**	Sensory integration/APAs	M-DT
**C**	Further challenging of movement complexity through increased levels of difficulty, task variation by successively integrating the balance components, and increasing demands of multi-tasking (i.e. cognitive and motor secondary tasks are performed simultaneously).	**7**	Sensory integration/APAs/motor agility/stability limits	C + M-DT
		**8**	Sensory integration/APAs/motor agility/stability limits	C + M-DT
		**9**	Sensory integration/APAs/motor agility/stability limits	C + M-DT
		**10**	Sensory integration/APAs/motor agility/stability limits	C + M-DT

The balance program divided into three blocks (Blocks A-C), with training principles and objectives for each block.

APAs = anticipatory postural adjustments; C-DT = cognitive dual-task training; M-DT = motor dual-task training; C + M-DT = mixed cognitive and motor dual-task training.

#### *Specificit*y

In contrast to non-specific training in PD [17], King and Horak [25] recently proposed a symptom-specific training approach targeting particular mobility impairments in individuals with PD. Our approach towards balance training resembles theirs regarding the principle of specificity. In the proposed intervention, we will use four balance components (sensory integration, APAs, motor agility, and stability limits) to target symptom-specific balance impairments associated with instability and falls in PD (see Table 1). The applications of these balance components will be separated according to exercise principles and objectives.

#### Varied practice

Through the course of training, exercise variation will be emphasised to enhance motor learning and promote generalisation of balance skills to everyday activities [43]. Based on previous findings, individuals with PD, compared with healthy individuals, need more time to achieve motor learning [50,51]. Therefore, as illustrated in Table 2, variation in the training will be increased successively through the course of training. During the initial phase of training (Block A), the exercise for each balance component will be trained separately in blocks on a weekly basis, to encourage familiarity of the principles and objectives and promote task-specific motor learning. In the later phases of training (Blocks B and C), generalisation of motor skills will be facilitated by increasing training variation with regard to characteristics of exercise in each balance component (e.g. variation in terms of body position, BoS and movement direction/velocity/amplitude) in Block B, and by integrating exercises from different balance components in Block C. In addition, multi-task exercises in Blocks B and C will further increase training variation (see Table 2).

#### Progressive overload

Based on previous recommendations [17] and national guidelines for physical training [52], as well as our experience from a pilot study of balance training in PD (data not yet published) and balance training in elderly [53], we believe that the training frequency, duration and total training dose planned for use in the proposed study (three 1-hour sessions/week during 10 weeks) will be sufficient to improve balance performance in elderly with mild to moderate PD. To achieve optimised challenges to individual capacities, the physiotherapists will individually adjust the difficulty level of the exercises for each balance component as follows:

*Sensory integration:* increased surface unevenness and restricted field of vision (vision-restricting glasses, carrying an object)

*APAs:* increased movement amplitude and velocity

*Motor agility:* increased gait complexity (e.g. altering velocity, step patterns, etc.), increased gait demands according to planned (awareness of upcoming sequences/tasks) and unplanned shifts upon verbal commands (unawareness of upcoming sequences/tasks) between different gait conditions, reciprocal movements and walking directions

*Stability limits:* changing the area or condition of the BoS and increasing leaning movement amplitude

Intermittent presence of reactive postural adjustments will be used as an indicator of the appropriate difficulty level. To target multi-task performance in PD, cognitive (e.g. counting, remembering items/numbers) and secondary motor tasks (e.g. carrying and/or manipulating objects) [54], will be introduced in Block B and further progressed in Block C (see Table 2). Multi-task training will be individually adjusted for each participant. The level of difficulty for the secondary tasks (motor or cognitive) will be considered appropriate if the primary task is affected (e.g. decreased gait velocity or increased stride variation). Moreover, as described above, training demands will additionally be challenged by increasing variation through the course of the program.

#### Feedback

In individuals with PD, goal-oriented oral feedback that addresses information externally to the body, i.e. concerning the performance of the task in relation to the environment (rather than the position or motion of the body), has been reported to enhance walking [55] and balance performance [56]. In the proposed trial, oral feedback will be as simple as possible and be externally oriented to promote movement automaticity. Cueing strategies (visual or oral), as described by Nieuwboer et al. [57], will be used when needed for participants experiencing severe freezing through the course of training; however, they will not be used as a standard training component.

#### Training/intervention procedure

The balance training will be performed in groups of five to seven participants in the facilities of the Physiotherapy Clinic at Karolinska University Hospital. To secure progression and safety, all training sessions will be led by two physiotherapists with experience of working with PD and elderly patients. To standardise the intervention and secure adherence to the proposed theoretical framework, all physiotherapists will participate in a theoretical and practical workshop before the intervention starts and will be supervised during the course of the training.

Every training session will start with a warm-up session for 5 min, consisting of varied walking tasks aiming to boost the cardiovascular system. The following 50 min (short rests included) will focus on highly challenging exercise blocks (approximately 10 min per block) of standing and walking conditions. The balance components of stability limits and APAs will mainly be addressed through standing exercises, and the balance components of motor agility and sensory integration will mainly be addressed in walking exercise conditions.

The program will end with a 5-min cool-down session with slow walking, axial stretching and breathing exercises. The number of exercise blocks for each training occasion and the distribution between standing and walking exercises will be adjusted to the level of ability in each group. After each training occasion, individuals’ performance of the exercises included will be briefly documented, as an evaluation of both the appropriateness of exercises and the current level of difficulty. Adverse events (defined as an injury or medical event that restricts everyday activities and participation in the intervention) and training adherence will be monitored and recorded [58].

#### Exercise on prescription

We predict that increased physical functioning following training will lead to greater confidence in everyday participation and therefore an increased level of physical activity. However, targeted advice is needed for long-term behavioural change [59,60]. Therefore, a meeting will take place after the last training session, where the participants will participate in a group discussion, led by physiotherapists, on how to sustain or increase their level of physical activity. Participants will be given Physical Activity on Prescription (PaP) [59,60], together with a leaflet on benefits of physical activity and health in PD.

### Control group

The subjects in the control group will receive usual care from their medical practitioner and/or the community services, and will not be restricted in participating in ongoing rehabilitation programs. The control group will be offered the same balance program after study termination.

### Outcome measures and test procedure

According to the wide disability spectrum associated with balance impairments in PD [16,61], a multi-dimensional battery of outcomes will be used to identify potential training effects. As illustrated in Table 3, we have applied the International Classification of Functioning, Disability and Health (ICF) as a conceptual framework to target relevant constructs of *body functions* (i.e. physiological functions of body systems), *activity* (i.e. execution of a task or action by an individual) and *participation* (i.e. involvement in a life situation) [62]. As previously suggested by Dibble et al. [16], our outcome measures represent a wide spectrum within the concepts of disability and health, from laboratory measurement of balance and gait performance, to free-living assessment of physical activity and self-perceived domains of QoL and participation in everyday living (see Table 3). Primary outcome measures will be functional balance performance, change in gait velocity (m/s) between single and dual task walking, and fear of falling. 

**Table 3:** **Classification of outcome measuresusing the International Classificationof Functioning, Disability andHealth (ICF)**

		**ICF**		
**Instruments**	**Domains**	Body functions	Activity	Participation
*Laboratory tests*				
- Electronic walkway^*^	Temporal and spatial gait parameters during single/multi-tasking conditions	X	X	
- Mod-CTSIB	Sensory integration	X		
*Clinical tests*				
- Mini-BESTest	Subdomains of balance performance	X	X	
- One-leg stance test	Balance performance, standing on one leg		X	
- Modified figure of eight test	Balance performance, walking in a figure of eight with reduced BoS		X	
*Free-living assessment*				
- Accelerometer	Physical activity		X	
*Questionnaires*				
- FES-I	Fear of falling		X	
- UPDRS ADL	Activities of daily living		X	
- SF-36	Generic health-related QoL			X
- PDQ-39	PD-specific health-related QoL			X

^*^GAITRite, CIR Systems Inc., Clifton, NJ, USA.

BoS = base of support; PD = Parkinson’s disease; QoL = quality of life.

FES-I = Fall Efficacy Scale International; Mini-BESTest *=* Mini Balance Evaluation Systems Test; Mod-CTSIB = Modified Clinical Test of Sensory Interaction and Balance; PDQ-39 = Parkinson’s Disease Questionnaire; SF-36 = 36-item Short-Form Health Survey; UPDRS ADL = the activities of daily living (ADL) section of the Unified. Parkinson’s Disease Rating Scale, part II.

Participants in both the intervention and the control group will be tested prior to and after the 10-week intervention, and at 9 and 15 months’ follow-up after entering the study. All physical measurements will be conducted during the ON phase of their levodopa medication and at the same time of day to restrict the influence of medication fluctuations. The order of the physical tests (laboratory and clinical, as described below) will be randomised for each participant to avoid systematic bias.

#### Laboratory tests

*Temporal and spatial gaitparameters* will be measured using a 9 m electronic walkway (GAITRite, CIR Systems Inc., Clifton, NJ, USA) sensing the geometry of each step through embedded pressure sensors. Four types of gait conditions will be evaluated during (i) self-selected and (ii) fast speed, (iii) while performing a cognitive (reciting every second letter of the alphabet) and (iv) a motor dual-task (carrying a tray with a glass of water). Spatial and temporal gait parameters will be analysed, as will variability and asymmetry in gait performance.

The *Modified Clinical Test ofSensory Interaction and Balance* (Mod-CTSIB) will be used to measure sensory integration during static standing [63]. To discriminate between the sensory systems (somatosensory, visual and vestibular), participants will be timed while standing on a force plate (50 cm x 50 cm; Kistler, Winterthur, Switzerland) in four different sensory conditions: (i) standing on a firm surface with the eyes open; (ii) standing on a firm surface with the eyes closed; (iii) standing on a compliant surface with the eyes open; and (iv) standing on a compliant surface with the eyes closed. Outcomes will be the time able to stand (max 30 s) and postural sway in the anterior-posterior and medio-lateral direction.

#### Clinical tests

The *Mini Balance Evaluation SystemsTest* (Mini-BESTest) will be used to assess underlying subsystems of balance performance (APAs, postural reactive responses, sensory integration and dynamic gait) [64].

The *One-leg stance test* will be performed with the subject standing on one leg as long as they are able to [65]. This test requires appropriate APAs for CoM transition from a standing position to a restricted BoS.

The *Modified figure of eighttest* will be used to evaluate dynamic balance during walking [66]. The subject is instructed to walk in a figure of eight (marked with 40 mm-wide tape on the floor, each loop having an internal diameter of 1.63 m) as fast as possible, with every step touching the tape [66].

#### Free-living assessment

*Physical activity* in everyday life will be assessed with accelerometers (Actigraph GT3X+, Manufacturing Technology Inc., Fort Walton Beach, FL, USA). The accelerometer will be used under free-living conditions, worn around the waist, using an elasticized belt, for 7 consecutive days. Outcomes will be total physical activity, expressed as counts per minute and steps per day, and time spent on different intensity levels as well as time spent sedentary [67].

#### Questionnaires

The Activities of Daily Living section of the *Unified Parkinson’s Disease RatingScale (UPDRS ADL)*, part II, will be used to measure activities of daily living [68].

The *Falls Efficacy Scale International-I* (FES-I) will be used to measure participants concern of fear of falling during a variety everyday activities (e.g. getting dressed, taking a shower, climbing stairs or going to the shop) [69].

As previously suggested by Brown et al. (2009), assessment of *Health-related QoL* in PD should include both a generic and a PD-specific instrument [70]. The Short Form-36 (SF-36) will be used to evaluate generic health-related QoL [70,71] and the 39-item Parkinson’s Disease Questionnaire (PDQ-39) will be used to evaluate PD-specific health-related QoL [70,72].

### Statistical analysis

Descriptive statistics will be performed to ensure comparability between scores at baseline as well as to test the assumption of homogeneity of variance for use of parametric statistics. To evaluate the effect of the intervention, mixed models (SPSS Inc., Chicago, IL, USA) with an alpha level of 0.05 will be used where appropriate. Significance of main or interaction effects will be explored using the Bonferroni post hoc test. In case of skewed distributed data, logarithmic transformations or corresponding non-parametric statistics will be used to assess main effects of the intervention.

### Sample size

The sample size for this study was determined from a pilot study (not yet published), including five mild to moderate PD subjects who participated in the proposed balance training, and also from previous trials on patients with PD and elderly people [53,73,74]. For each sample size calculation, power was set at 80% and a two-sided test at the alpha level of 5%. For the Mini-BESTest, a sample size of 24 was required to detect a 3-point difference between groups, assuming a mean variance or standard deviation (SD) of 3.6 (effect size: 0.83). For the interference effect of dual task performance, i.e. percent change in gait velocity (m/s) between single and dual task walking [74], a sample size of 27 subjects was required to detect a difference of 10% (SD 13) between groups (effect size = 0.77). Finally, for the FES- I, a sample size of 32 subjects was required to detect a difference of 2 points (SD 2.8) between groups (effect size = 0.71). The dropout rate was set at 15%, corresponding to intervention studies with PD subjects [75,76]. Taken together, to ensure adequate statistical power the sample size for this study would optimally be 40 per group (total n = 80).

## Discussion

In this study protocol, a conceptual framework based on current scientific knowledge from neuroscience constraints in PD is used to design a training intervention targeting balance and walking performance in PD. The rationale for this training approach relies on findings of preserved motor learning in mild to moderate stages of PD [51] and the plausibility of training-induced neuroprotection [14,15]. Although previous exercise interventions in PD have reported promising results for balance and gait performance [16-18], they often lack to report the underlying principles of the intervention [21]. In particular, knowledge concerning the influence of active elements of the intervention (e.g. exercise components, training dosage and intensity) is limited [21]. In contrast to the majority of previous training regimes that emphasise general physical training [17] or target single physiological systems (e.g. muscular strength, reactive postural adjustments), the active elements in this intervention cover traditional training principles of specificity, progressive overload and random training. Hence, these principles are modified to promote motor learning in PD through specific balance components related to PD symptoms during highly challenging and varying exercise conditions. There is an urgent need to investigate both short-term and long-term effects of new exercise strategies targeting balance control in PD [17].

Balance impairment in PD comprises a wide spectrum of disability and health [16]. Detailed long-term follow-up, 1 year post-intervention, in the proposed study will give a unique opportunity to address important questions concerning potential effects of and limitations for group-based balance training. First of all, the proposed study will be one of the first trials to provide knowledge of potential effects of exercise intervention according to the whole spectra of ICF domains, including laboratory measurements, clinical tests, and free-living and self-perceived assessments (see Table 3). Such knowledge will be essential for designing and improving exercise interventions in the future. Secondly, long-term follow-up will provide knowledge of the maintenance of potential training effects. This is particularly important for PD given the progressive deterioration of the disease in which regular periods of supervised balance training may be required to sustain physical performance.

Since this intervention will not be restricted to a fixed program with specific exercises, several steps will need to be taken to standardise the intervention by educating physiotherapists in the framework and implementation of the training approach, as well as supervising the physiotherapists during the course of training and monitoring training performance as well as progression. During the course of training, each training session is planned with regard to the different phases of the intervention (see Table 2) and adapted by the physiotherapist to meet individual capacities of the participants. The physiotherapists conducting the training will be responsible for transferring the conceptual framework into practice, using a pragmatic approach rather than restricting the intervention to a standardised protocol. Thus, translation of this research intervention into the clinical setting will be enhanced.

There are several limitations to the proposed study. The result of this intervention can only be generalised to individuals with mild to moderate PD [45] and group training in outpatient care. All measurements will be conducted at the same time of the day during the ON phase of levodopa medication. The total dose of medication will be monitored in a longitudinal perspective [77], though separating the potential effects of balance training from those of levodopa medication will be limited. Moreover, blinding of the test leaders will not be possible since they also will serve as support for the physiotherapists conducting the intervention. However, this proposed randomised controlled trial has the potential to provide new knowledge and a change in focus concerning balance interventions in PD.

## Competing interests

The authors declare that they have no competing interests.

## Authors’ contributions

AS and EF contributed to the research design and obtained funding for the study. DC, EF and NL provided the design of the study, intervention and outcome measures. MH contributed with design of the Physical Activity on Prescription-part of the intervention. DC was principally responsible for the drafting of the manuscript. All authors critically reviewed the manuscript and approved the submitted version.

## Pre-publication history

The pre-publication history for this paper can be accessed here:

http://www.biomedcentral.com/1471-2377/12/111/prepub
